# Refractive Error and Ocular Pathology of Children Examined in an Ophthalmological Practice in Moldova

**DOI:** 10.3390/jcm14051554

**Published:** 2025-02-26

**Authors:** Veronica Ziziuchin, Gro Horgen, Vibeke Sundling

**Affiliations:** 1 National Centre of Optics, Vision and Eye Care, Department of Optometry, Radiography and Lighting Design, University of South-Eastern Norway, 3616 Kongsberg, Norway; gro.horgen@usn.no (G.H.); vibeke.sundling@usn.no (V.S.); 2 Department of Ophthalmology, TMA Ciocana Clinic, 2052 Chisinau, Moldova

**Keywords:** hyperopia, myopia, eye disease, children, vision, ophthalmological practice

## Abstract

**Background/Objective:** Poor vision can lead to low academic performance and negatively affect the quality of life. In Moldova, there are few guidelines for vision and eye care in children. Further, the prevalence of refractive errors, visual impairment, or eye disease in Moldovan children is unknown. The study aimed to explore the prevalence of refractive errors and eye disease among children examined in ophthalmological practice in Moldova. **Methods:** The study had a cross-sectional design, including consecutive patients, aged <18 years, examined in an ophthalmological practice in Chișinău, Moldova, during two time periods, from July to September 2018 and from May to June 2023. **Results:** Data of 299 children (157 female) aged 0–18 years were collected. In all, 177 (59.2%) children had cycloplegic refraction and assessment of refractive error: 47 in the age group 0–2 years, 64 in the age group 3–6 years, and 66 in the age group 7–18 years. In children under 3 years, 8 (17%) were emmetropic (−0.50 D < SER < +0.50 D), 4 (9%) were myopic (SER ≤ −0.50 D), 34 (72%) had mild hyperopia (0.5 ≤ SE < 3.0), and 1 (2%) had high hyperopia (SER > 3.0). Among children aged 3–6 years, 11 (17%) were emmetropic (−0.50 D < SER < +0.50 D), 5 (8%) were myopic (SER ≤ −0.50 D), 46 (72%) had mild hyperopia (0.5 ≤ SER < 3.0), and 2 (3%) had high hyperopia (SER > 3.0). In children over 6 years, 33 (50%) were myopic (SER ≤ −0.50 D), 2 (3%) were emmetropic (−0.50 D < SER < +0.50 D), 27 (41%) had mild hyperopia (0.5 ≤ SER < 3.0), and 4 (6%) had high hyperopia (SER > 3.0). A total of 4 children (2.3%) had amblyopia and 19 children (6.4%) had ocular pathologies. Twelve children (6.8%) were visually impaired with their habitual correction. **Conclusions:** Half of the school children had myopia and a little less than half had hyperopia. Among toddlers and pre-school children, one in four had myopia or were at risk of developing myopia.

## 1. Background

Refractive errors result in blurred vision and eye strain impacting successful education and social integration [1,2,3,4]. Children’s school performance relies on good vision [5,6], and the level of education influences job opportunities and the personal economy. Refractive errors also have a significant economic impact on society: The global costs of myopia are several hundred billion dollars annually, including both direct health expenditure and indirect costs such as lost productivity [7]. Myopia (in particular, but also other refractive errors) cause additional costs because of related complications. Because of eye elongation, myopia leads to retinal changes that may result in uncorrectable visual impairment [8]. Hypermetropia, on the other hand, is associated with an increased risk of amblyopia and esotropia [9], and may affect reading and academic performance in children and youth [4,5,6]. Importantly, uncomplicated, uncorrected refractive errors are one of the leading causes of vision impairment worldwide [10], which can be easily avoided with low costs. According to the National Advisory Eye Council, the yearly cost of correcting refractive errors with spectacles or contact lenses in the USA is only USD 2 billion. In the USA, 84.5% of the population has refractive errors [11]. Myopia ranges from 10% in Caucasians [12,13] to 90% in Asians [14]. In Nordic countries hypermetropia prevails over myopia [15,16]. These differences may be caused by genetics [17,18], but also by environmental factors and lifestyle [19,20,21] that may influence gene expression [22,23].

Knowledge about people’s eye health and vision is important for adequate development of public health policies [24]. In Moldova, this knowledge is sparse. The reported prevalence of blindness and visual impairment in people aged ≥50 years are 1.4% and 2.2%, respectively [25]. The prevalence of refractive errors and eye disease in Moldovan children is not known. Although all children undergo routine eye examinations at 3 months, and at 1, 3, and 6 years as a part of the national health care system, standardised protocols and national guidelines for eye and vision examination in children are limited. As a first step to gaining knowledge about children’s vision and informing policymakers, this study explores the prevalence of refractive error and eye disease in children examined in Moldovan ophthalmological practice.

## 2. Methods

### 2.1. Study Population and Recruitment

The study had a cross-sectional design exploring refractive error and ocular disease among children (0 to 18 years) in Moldova. The target population was patients examined in an ophthalmological practice in Chișinău, Moldova. Chișinău is the capital of Moldova, and the largest city of Moldova. Further, it is the main industrial and commercial centre, and situated in the middle of the country.

The gender distribution of the child population in Chișinău at the beginning of 2018 year is presented in Table S1 and the distribution of ethnicities in Chișinău versus Moldova is presented in Table S2. We did not collect information about distribution of ethnicities nor family economic status of the children in the study.

The sample population consisted of consecutive patients examined by one ophthalmologist (VZ) in one ophthalmological practice (MSPI TMA CIOCANA) during two time periods, from July to September 2018 and from May to June 2023. All patients attending the clinic during this period were invited to participate in the study.

### 2.2. Data Collection

The ophthalmological assessment was undertaken according to tacit national consensus, patients’ complaints and diagnosis, and the national guidelines for vision and eye examination in children [26,27,28]. The examination included habitual visual acuity (HVA), autorefractor keratometry (HRK 7000 A, Huvitz, Anyang, Gyeonggi-do, Republic of Korea), retinoscopy, subjective refraction, best corrected visual acuity (BCVA), slit lamp examination, and ophthalmoscopy.

Visual acuity was measured on a logMAR chart and the number of letters read defined the visual acuity.

Investigations were undertaken according to age and the ability of the child to cooperate during the examination. Distance retinoscopy was performed in all children aged > 8 months age. In 2018, the dry retinoscopy result guided the use of cycloplegic refraction. Cycloplegic refraction was not undertaken if dry retinoscopy was considered within normal age limits [29,30], according to Moldovan regulations. Normal age limits of refractive error were defined for children < 3 years as mild hyperopia (spherical equivalent (SER) ≤ 2.75 D) and for children > 6 years as emmetropia (−0.50 D ≤ SER ≤ +0.50 D). In 2023, all children underwent cycloplegic refraction. In children < 6 years, cycloplegic refraction was performed using one drop of Tropicamide 0.5%. In children ≥ 6 years, one drop of Tropicamide 1% was applied twice at 15 min intervals. In the analysis of refractive error in this study, children who did not undergo cycloplegic refraction were excluded.

Visual acuity, autorefractor keratometry, subjective refraction, slit lamp examination, and ophthalmoscopy were performed for children from 4 years of age. The eyelids and anterior segment were examined in all children using either slit lamp from 4 years of age and by direct examination with diffused light in children under 4 years old. The posterior segment was examined either by slit-lamp indirect ophthalmoscopy using a Volk 78D lens, Volk Optical Inc., Mentor, OH, USA, or by direct ophthalmoscopy using a Heine Beta 200 ophthalmoscope, HEINE Optotechnik GmbH & Co., Gilching, Germany. Diagnosis of ocular disease was made according to clinical judgement and guidelines [26,27,28].

### 2.3. Data Analysis

Data for refractive errors and ocular pathologies were analysed and presented at individual levels [31]. Refractive error was established as the mean of three autorefractometer measurements. We defined and classified refractive error by spherical equivalent refractive error (SER) (sphere + ½ cylinder power) as myopia (SER ≤ −0.50 D), emmetropia (−0.50 D < SER < +0.50 D), hyperopia (SER ≥ +0.50 D), and astigmatism (refraction ≥ 1.00 DC) [15,30]. Diagnosis of ocular pathologies was established and defined according to International Classification of Diseases 10th Revision (ICD-10) [32].

### 2.4. Ethics

The research followed the tenets of the Declaration of Helsinki. The research project was reviewed by the Regional Committees for Medical and Health Research Ethics (REK) and deemed outside the scope of the Health Research Act, and, therefore, not subject to approval. The project was approved by the clinic MSPI TMA CIOCANA and reviewed by the Norwegian Data Protection Services (848871). Participants and their caretakers received verbal and written information about the study before providing written, informed consent.

## 3. Results

**Figure 1:**
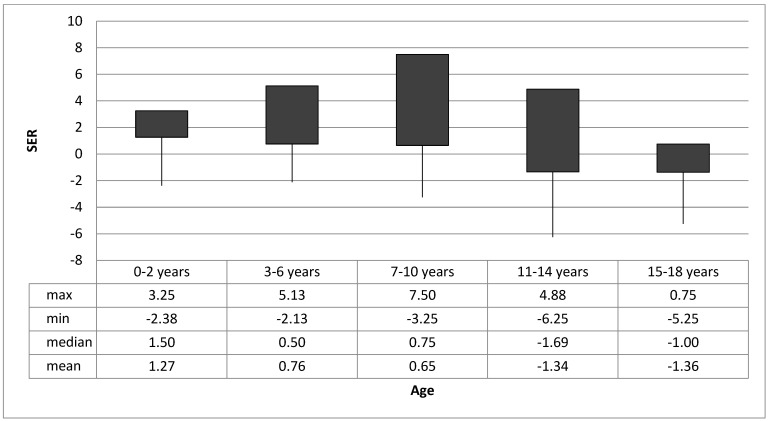
Refractive errors in age groups. Abbreviations: SER: spherical equivalent refraction.

In all, 299 children aged 0–18 years were examined during the two time periods. All children (*N* = 299) were European Caucasians, and 157 (52.5%) were female. A total of 177 (59.2%) had cycloplegic refraction and were included in the assessment of refractive error. A total of 47 of these children (26.5%) were aged 0–2 years, 64 (36.2%) were aged 3–6 years, 40 (22.6%) were aged 7–10 years, 16 (9.0%) were aged 11–14 years, and 10 (5.7%) were aged 15–18 years. In all, 107 (60%) of the children had mild hyperopia, 7 (4%) had high hyperopia, 42 (24%) had myopia, and 21 (12%) emmetropia; see Table 1. The refractive error for the different age groups is shown in Figure 1.

**Figure 2:**
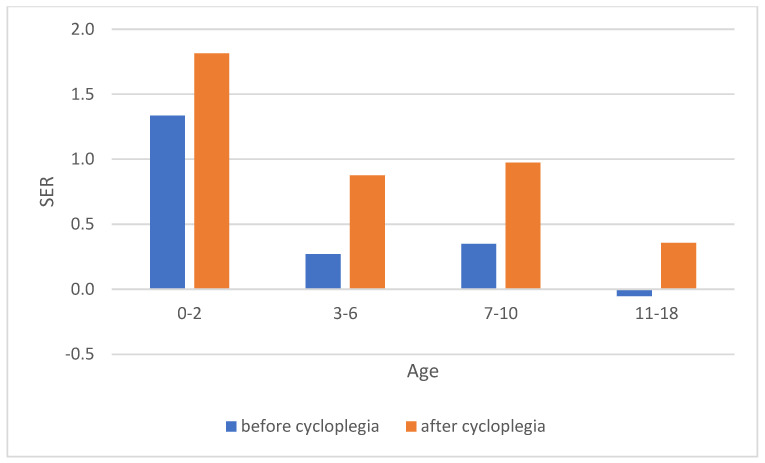
Refractive errors before and after cycloplegia. Abbreviations: SER: spherical equivalent refraction.

There was a significant difference of −0.55 D in mean refractive errors between boys (0.86 + −1.60) and girls (0.31 + −1.81), t(175) = 2.13, *p* = 0.035. Refractive errors was +0.54 more hyperopic after cycloplegia (1.21 + −1.46), compared to before (0.66 + −1.43), t(92) = 9.1, *p* < 0.0001. The difference between before and after cycloplegia in refraction was larger in the 7–10 age group and smaller in the 11–18 age group, and also smaller in the 0–2 and 3–6 age group compared to the 7–10 age group (Figure 2).

A total of 36 (20.3%) children, 18 girls and 18 boys, had astigmatism, and half of them were younger than 5 years of age. Four (2.3%) children had amblyopia: two had mild amblyopia (visual acuity of 6/9 to 6/12) and two had moderate amblyopia (worse than 6/12 to 6/36). The amblyopia was due to refractive anisometropia in all cases.

Ten children presented with mild visual impairment (6/18 < HVA < 6/12) and two children presented with moderate visual impairment (6/60 < HVA < 6/18). These children had not had adequate habitual optical correction or no optical correction. With appropriate optical correction, none were visually impaired.

Nineteen children (6.4%) were diagnosed with eye disease: seven (2.3%) had stenosis or insufficiency of lacrimal passages, three (1%) had retinopathy of prematurity, two (0.7%) had conjunctivitis, one (0.3%) had congenital cataracts, two (0.7%) had chalazion, two (0.7%) had strabismus, one (0.3%) coloboma of the iris, and one (0.3%) eyelid haemangioma.

## 4. Discussion

To the best of our knowledge, this is the first study of refractive errors and eye disease in Moldovan children. A study of vision and eye health in Moldova in 2012 only assessed prevalence of blindness and visual impairment in people aged ≥ 50 years [25].

Half of the schoolchildren examined in our study had myopia. The frequency is higher than the reported prevalence in Europe and lower than reported in Asia [33]. There is a trend of increasing myopia prevalence from Western to Eastern countries that may be explained by genetic factors [34]. However, there are studies reporting no differences in genetic myopia between Europeans and Asians [35], and the prevalence and progression of myopia is also associated with environmental factors (time spent outdoors), education, and personal characteristics [21].

Hence, the aetiology of myopia is still under debate, so is the link between sex and refractive error. Studies show that sex is a determinant factor for myopia [36] and it is associated with heterozygosity of common L opsin exon 3 haplotypes [37]. However, some studies show a strong correlation between female sex and myopia [38], whereas others studies report a higher myopia prevalence in boys [39]. In our study, myopia was more frequent in girls. Further, the prevalence of myopia increased with increasing age of children. This could be associated with lifestyle changes and spending more time indoors completing near work [19,40] using computers and smartphones [20] with age increasing. The myopia prevalence is higher in younger generations [14]. Lifestyle changes have led to an increase in myopia prevalence in recent decades, and there is expected to be a doubling of myopia prevalence globally by 2050 compared with 2000 [41].

Although the refraction of the majority of children under 7 years in our study was within normal age limits [29], 20–25% had myopia or were at risk of developing myopia. If not taken under control, children at risk will develop myopia, and the myopia will increase in those who already are myopes, and by that also the risk of ocular complications. Adequate myopia control will most likely prevent the increase in visual impairment in adults [8]. In light of the possible myopia epidemic, it is very important to ensure adequate eye care and policymakers need to make the right decisions [42].

While myopia is studied worldwide, lower attention is paid to hypermetropia, as it is considered normal within a certain age limits [30] and not as harmful as myopia, even though it may lead to amblyopia and esotropia [9], as well as reduced academic performance and level of education. As expected, in our study, mild hypermetropia decreased with age, but high hyperopia (SER ≥ +3.0) was found to be 6% in school age children. Two out of ten children who presented with mild visual impairment (6/18 < HVA < 6/12) with their habitual optical correction were hyperopes.

Despite the high number of children with refractive errors and that the impact of the uncorrected refractive error is known, there are still children with inadequate correction or lack of optical correction in Moldova.

The prevalence of ocular pathologies was 6.4%. These findings show a lower frequency of ocular pathologies compared with data from other studies from other parts of the world that found a prevalence of ocular pathologies about 20% in ophthalmic practice [43,44]. Some children came for a routine eye examination, without complaints, referred by the general practitioner, whereas others came with concrete complaints.

Taking into consideration that the majority of children who visited the ophthalmologic office needed only adequate optical correction and no other treatment, it is wise to consider task shifting and involve optometrists in childhood eye and vision care. In Moldova, a first step in this direction was taken in 2017 when the optometry education was established. The first optometrists graduated in Moldova in 2021; up until then, there were no optometrists in Moldova. According to Moldova’s regulations, optometrists perform a complete eye examination. They prescribe vision correction, but do not prescribe ocular therapeutics. When they detect an ocular pathology, they refer patients to an ophthalmologist. The new eye care profession entering the health care system provides new possibilities for establishing a national guideline for sustainable eye and vision care for children in Moldova.

This study has some limitations. The number of examined children is small and the study population is restricted to patients examined in a single ophthalmological practice by one ophthalmologist. This limits the generalizability to the general population of children in Moldova. To provide accurate estimates of prevalence, a larger random sample of Moldovan children should be examined in a population study. Further, cycloplegic refraction was performed with Tropicamide, which is the standard of care in Moldova.

The cycloplegic effect of cyclopentolate has been evaluated by the type of refractive errors, refractive assessment procedure, and age group, and found to be no different than Tropicamide in children, hyperopic patients, and by using retinoscopy [45]. Tropicamide can, therefore, be used for cycloplegia [46,47]. However, we are aware that the amount of cycloplegia can be variable [45] and may have influenced the refractive error reported in this study. The lack of using any cycloplegia is critiqued [48], as the prevalence of hyperopia and emmetropia is underestimated and the prevalence of myopia is overestimated [49]. In our study, there is a difference between the refraction before and after cycloplegia, an indication that a cycloplegic effect is achieved. The difference between before and after cycloplegia in refraction is larger in the 7–10 age group and smaller in the 11–18 age group, and this could be explained by the fact that the accommodation decreases with age. The difference is also smaller in the 0–2 and 3–6 age group compared to the 7–10 age group, and this is probably due to lower concentration of Tropicamide used for these age groups. Lack of using any cycloplegia can lead to unnecessary costs for myopia control in cases when it is not needed, and lack of adequate correction in children with hyperopia who are not detected [9].

## 5. Conclusions

Nine in ten children had some kind of refractive error. Half of the school-aged children had myopia and a little less than half had hyperopia. Among toddlers and pre-school children, one in four had myopia or were at risk of developing myopia. The prevalence and correction of refractive error in Moldovan children is of concern, and future population studies are required to provide knowledge for policymakers to ensure children’s vision and eye care.

## Figures and Tables

**Table 1:** Frequency of refractive errors, n (%) [95% CI].

	N	SER ≤ −0.5	−0.50 D < SER < +0.50 D	+0.50 D ≤ SER < +3.00 D	SER ≥ +3.0
All	177	42 (24%) [0.18–0.31]	21 (12%) [0.08–0.18]	107 (60%) [0.53–0.68]	7 (4%) [0.02–0.08]
0–2 years	47	4 (9%) [0.02–0.20]	8 (17%) [0.08–0.31]	34 (72%) [0.57–0.84]	1 (2%) [0.00–0.11]
3–6 years	64	5 (8%) [0.03–0.17]	11 (17%) [0.09–0.29]	46 (72%) [0.59–0.82]	2 (3%) [0.00–0.11]
7–18 years	66	33 (50%) [0.37–0.63]	2 (3%) [0.00–0.11]	27 (41%) [0.29–0.54]	4 (6%) [0.02–0.15]
*7–10 years*	40	12 (30%) [0.17–0.47]	2 (5%) [0.01–0.17]	23 (58%) [0.41–0.73]	3 (7%) [0.02–0.20]
*11–14* *ars*	16	13 (81%) [0.54–0.96]	0	2 (13%) [0.02–0.38]	1 (6%) [0.00–0.30]
*15–18 years*	10	8 (80%) [0.44–0.97]	0	2 (20%) [0.03–0.57]	0

Abbreviations: SER: spherical equivalent refraction.

## Data Availability

The raw data supporting the conclusions of this article will be made available by the authors on request.

## Supplementary Materials

The following supporting information can be downloaded at: https://www.mdpi.com/article/10.3390/jcm14051554/s1, Table S1: Gender distribution of children population in Chisinau vs study population, at the beginning of 2018 year. Table S2: Distribution of ethnicities in Chisinau and total in Moldova, 2014.
