# Supplementary material for: Refractive Error and Ocular Pathology of Children Examined in an Ophthalmological Practice in Moldova
Source: J Clin Med. 2025 Feb 26;14(5):1554. doi: 10.3390/jcm14051554 (PMC11899808; doi:10.3390/jcm14051554)
Supplement: Supplementary file 1 [file jcm-14-01554-s001.zip › jcm-3350438-supplementary.pdf]

Table S1. Gender distribution of children population in Chisinau vs study population, at the beginning of 2018 year

	Chisinau population		Study population	
	male	female	male	female
0-2 years	15.50%	15.20%	59.79%	60.75%
3-6 years	22.83%	23.19%	19.59%	12.15%
7-17 years	61.66%	61.61%	20.62%	27.10%

Table S2. Distribution of ethnicities in Chisinau and total in Moldova, 2014

Ethnic group	Chisinau	Moldova
Moldovans	67.18	75.00
Romanians	14.46	7.00
Ukrainians	5.95	6.57
Gagauzians	0.68	4.57
Russians	9.29	4.06
Bulgarians	1.07	1.88
others	1.37	0.92
